# Q: Is Addiction a Brain Disease or a Moral Failing? A: Neither

**DOI:** 10.1007/s12152-016-9289-0

**Published:** 2017-05-18

**Authors:** Nick Heather

**Affiliations:** Department of Psychology, Northumbria University, 8 Roseworth Terrace, Newcastle on Tyne, NE3 1LU UK

**Keywords:** Brain disease model of addiction, Moral model, Developmental-learning model, Addiction as a disorder of choice, Public-facing communications, Stigma

## Abstract

This article uses Marc Lewis’ work as a springboard to discuss the socio-political context of the brain disease model of addiction (BDMA). The claim that promotion of the BDMA is the only way the general public can be persuaded to withhold blame and punishment from addicts is critically examined. After a discussion of public understandings of the disease concept of addiction, it is pointed out that it is possible to develop a scientific account of addiction which is neither a disease nor a moral model but which the public could understand. Evidence is reviewed to suggest that public acceptance of the disease concept is largely lip-service and that the claim the BDMA removes stigma among the public and professionals is unsupported by evidence. Further, there is good evidence that biogenetic explanations of mental/behavioural disorders in general have been counterproductive in the attempt to ally stigma. A model of addiction as a disorder of choice may attract special problems in public-facing communications and risks being misunderstood. However, ways of presenting this model to the public are suggested that may avoid such risks. Lastly, the claim that the BDMA is the only way of ensuring access to treatment and of maintaining research funding for addiction is disputed and a way in which these benefits can be retained under a disorder-of-choice model proposed. The article concludes by enthusiastically endorsing Lewis’ call for a third stage in the governing image of addiction.

## Introduction

Marc Lewis is unusual, perhaps unique,^1^ in being a neuroscientist interested in addiction who believes that it is not best seen as a brain disease [3–5]. He has done a great service to those of us who share his view that the brain disease model of addiction (BDMA) is mistaken but who lack the expertise to criticise it on neurobiological grounds. But despite this debt of gratitude, I will not be concerned in this article with Lewis’ analysis of where the BDMA goes theoretically wrong or with the internal inadequacies of any other disease account of addiction (but see [6–8]). Rather, I want to use Lewis’ work as a springboard to discuss the socio-political context of the BDMA and its possible alternatives and, specifically, a spurious claim often made by BDMA supporters.

The claim is that the promotion of the BDMA is the only way in which members of the general public, and presumably policy-makers, opinions-formers, etc. too, can be persuaded to withhold blame and punishment from addicts for their troublesome behaviour and the only way to combat the stigma society attaches to them. Put another way, the claim is that a rejection of the BDMA is equivalent to believing and promoting the idea that addiction signifies a moral failing on the part of addicts. This follows from the assumption that addiction is either a brain disease or a moral failing, with no other understanding of addiction possible.

Many examples of this claim could be provided but I will focus here on two – from the early days of the attempted popularisation of the BDMA and from the latest defence of the model by its most prominent supporters. In what may be termed the BDMA manifesto, Alan Leshner, then Director of the *National Institute on Drug Abuse* (NIDA) in the USA, wrote [9] as follows:

“One major barrier (to closing the gap between scientific and public understanding) is the tremendous stigma attached to being a drug user or, worse, an addict. The most beneficent public view of drug addicts is as victims of their societal situation. However, the more common view is that drug addicts are weak or bad people, unwilling to lead moral lives and to control their behavior and gratifications. To the contrary, addiction is actually a chronic, relapsing illness, characterized by compulsive drug seeking and use …. The gulf in implications between the ‘bad person’ view and the ‘chronic illness sufferer’ view is tremendous. As just one example, there are many people who believe that addicted individuals do not even deserve treatment. This stigma, and the underlying moralistic tone, is a significant overlay on all decisions that relate to drug use and drug users” (p.45, parentheses added).

The implication of this passage is that the only alternative to seeing addicts as weak or bad people, and thus the only way to avoid stigmatising them, is to view them as suffering from a chronic, relapsing (brain) illness.

In a recent article aimed at defending the BDMA against critics, Nora Volkow, the current Director of NIDA, together with two prominent supporters of the BDMA [10] insists that “After centuries of efforts to reduce addiction and its related costs by punishing addictive behaviors failed to produce adequate results, recent basic and clinical research has provided clear evidence that addiction might be better considered and treated as an acquired disease of the brain” (p.363). The implication here is that it is only the discovery that addiction is a brain disease that has put an end to the punishment of addicts. They go on:

“The concept of addiction as a disease of the brain challenges deeply ingrained values about self-determination and personal responsibility that frame drug use as a voluntary, hedonistic act. In this view, addiction results from the repetition of voluntary behaviors. How, then, can it be the result of a disease process? The concept of addiction as a brain disease has even more disconcerting implications for public attitudes and policies toward the addict. This concept of addiction appears to some to excuse personal irresponsibility and criminal acts instead of punishing harmful and often illegal behaviors” (p.364).

The influence of the article by Volkow and colleagues, published in a prestigious medical journal, quickly found its way into the blogosphere. The journalist Tori Utley, writing for *Forbes,* was convinced by the article that “addiction should be viewed as an acquired disease of the brain, based on research that continues to give neurobiological evidence of addiction as a disease, not a moral failing” [11]. Dr. Michael Brant-Zawadzki, executive medical director of the *Hoag Neurosciences Institute* in California, went further in asserting that labelling addiction as a choice or habit, instead of “what it truly is”, a chronic treatable brain disease, is intellectually dishonest [12].

Enough has been said to establish that the promotion of the BDMA is typically accompanied by the rider that its only alternative is a ‘moral’ view of addiction.

## What Does the Public Understand by the Disease of Addiction?

There has been too little research on this important question but some answers to it can be advanced. It is clear that the meaning of the term in the ordinary language has changed significantly over the centuries [13] and that more recent usage has expanded from psychoactive substances to a wide range of substance-related and non-substance activities, to the point arguably where ‘addiction’ signifies little more than something a person spends a great deal of time doing [14].

In terms of attributed causality, Robin Room [15] has suggested that the addiction concept offers modern society “a secular equivalent for possession as an explanation of how a good person can behave badly, and as an inner demon over which a hero can triumph” (p. 221). If so, it should be noted that, like the concept of demonic possession, this attempted explanation is circular: Why do people behave repeatedly in ways that are bad for them? Because of addiction; What is addiction? Behaving repeatedly in ways that are bad for you.

Whatever its explanatory function as a cultural concept, however, it seems true that, in relation to substance use if nothing else, addiction *can* serve the purpose in society that supporters of the BDMA claim for it - providing a way for members of the general public and others to accept, superficially at least, that addicts should not be punished for their troublesome behaviour but should instead receive compassion and care. Indeed, the disease concept has been promoted from about the end of World War 2 as a mark of liberal and enlightened opinion. In contrast to the old-fashioned and reactionary view that addicts are, in Leshner’s words, weak or bad people, it is now known that addicts ‘can’t help’ behaving the way they do because they are compelled to behave that way by their disease of addiction. This exculpatory logic applies, of course, to any disease model of addiction of which there are a great many, but seems to have been taken over by supporters of the BDMA as though they were the first to think of it. Conversation with a member of the public, or journalist, politician or nearly anyone who claims to believe that addiction is a disease, reveals that this is *all* they mean by it; further inquiry soon results in the kind of circular reasoning noted above. Room may be right about the deeper cultural function of the disease concept but, in terms of practical consequences, all it seems to mean for those members of the public who subscribe to it is that addictive behaviour is caused by some, usually unspecified kind of disease and that, as a result, addicts shouldn’t be blamed or punished for behaving as they do.

Let us assume for the purposes of the remainder of this article that Marc Lewis is correct and that the BDMA is fundamentally flawed in ascribing to addiction a disease status based on a neurological abnormality. Let us also assume that, to make progress in alleviating the harmful effects of addictive behaviour, it is necessary to develop, test and refine some other account of addiction – Lewis’ own developmental-learning model or some other model that discards the foundational premise of addiction as a disease. Would such an account inevitably sow confusion in the public mind? Would rejection of the disease concept lead inexorably, as supporters of the BDMA perspective would very likely claim, to a reversion to moralistic attitudes and punitive responses to addiction? Thus the question to be addressed in this article is whether it is true that there is no other way to persuade the public that addicts should receive sympathy and, when they ask for it, help to change their behaviour. Related questions are whether the disease concept in general and the BDMA in particular is successful in reducing stigma and whether there is no other way of doing so.

## Addiction and Science in the Public Understanding

The idea that calling it a disease is the only understanding of addiction that avoids blaming and punishing addicts is simply untrue. It is obviously possible to present a behavioural, a psychosocial, a biopsychosocial or, indeed, any other plausible model of addiction without implying either that it is a disease or that addicts should be blamed and punished. For example, while Lewis [3] generously concedes that the disease model “has countered the perception that addicts are morally deficient or self-indulgent, arguably reducing the stress and isolation they and their families experience” (p. 7), there is absolutely nothing in his own developmental-learning model that, despite its rejection of the disease model, leads us to conclude that we should return to seeing addicts as morally deficient or self-indulgent. It should go without saying that one can’t derive an ‘ought’ from an ‘is’ and that blame and punishment have no place in science. A modern alternative to a moralistic view of addiction is a scientific view but disease models of addiction, including the BDMA, represent only one subset of possible scientific accounts. So, in claiming that it is only by seeing addiction as a brain disease that blame and punishment can be avoided, Leshner, Volkow and their colleagues, in the remarks quoted above, confuse ‘disease’ and ‘science’.

It might be claimed, nevertheless, that the attribution of disease is the only way the general public can *understand* the proposition that addicts should not be held morally accountable for their behaviour – the only way, in other words, that addiction can be apprehended as being *caused* rather than freely chosen. Such a view would be deeply patronising. When the first modern disease concept of alcoholism was formulated in the 1940s and 1950s (see [7]), the only mature, scientific language readily available for the public to approach an understanding of addiction came from medical science (apart perhaps from psychoanalysis, which has never had much to offer an understanding of addiction). Now, however, there is a much wider appreciation of concepts derived from neurological, behavioural and social science that can be used for this purpose and can avoid the conflation of cause and disease. With the continuing expansion of higher education and growing participation at various levels of specialism on courses on aspects of neurology, psychology and sociology, the public is much more *au fait* with concepts and principles that would allow them to follow non-disease explanations of addiction. Some kind of test of this assertion would be to follow up the response to articles and interviews in popular newspapers and magazines in which Marc Lewis has tried to explain his theory of addiction to the public [e.g., 16–18].^2^

## Stigma

As Marc Lewis would agree, the disease concept has been responsible over the years for diverting many users of illicit drugs and addicts from prison to treatment and this is no mean achievement. A wider claim, as we have seen, is that, because addicts are not held responsible for their behavior, the disease concept can remove the stigma that would otherwise be attached to addiction in the public mind. Is this true?

The first point to make is that the interplay between addiction and stigma is more complicated than this question suggests. First, general disapproval of the effects of heavy drinking or other substance use is an important informal social control that helps to inhibit the progression to heavier use and more serious harm [19]. Although society’s attitudes to drug use and intoxication are fundamentally ambivalent [20], these informal social controls must be counted a form of stigma but one presumably with benign effects. Any attempt to remove stigma from drug use and addiction, not merely one based on the disease concept, must take account of this inevitable barrier.

Secondly, the straightforward proposal that seeing a behavior as the consequence of disease necessarily leads to benign attitudes to it can be empirically examined and is, in fact, found considerably wanting. Some 30 years ago, John Crawford and I carried out a survey of the general public in Scotland regarding their attitudes to the disease concept of alcoholism and the treatment of alcoholics [21]. Although concerned with only one kind of addiction, the findings of this survey are directly relevant to the issues under discussion here and have not received the attention they deserve. We gave a representative sample of 200 members of the general public in Dundee a questionnaire on beliefs about alcoholism and attitudes to alcoholics. We then subjected responses on 5-point Likert scales to factor analysis.

The results of this survey showed first that the majority of the public (70 %) endorsed the disease concept by agreeing that “Alcoholism is best seen as a form of disease or illness”. This finding was comparable to figures reported in the American attitudes literature up to that time and suggests that, even 30 years ago and before the BDMA had been promoted, the widespread publicity campaign to have alcoholism recognized as a disease had ostensibly met with success, at least in the north-east of Scotland. However, and this is the crucial point, the factor analysis showed that whether or not respondents endorsed the disease concept of alcoholism had little directly to do with whether or not they had a sympathetic attitude to the treatment of alcoholics and believed that public money should be devoted to treatment, etc.; the latter was better predicted by more general, non-condemnatory and humanitarian attitudes to socially deviant groups as a whole, an attitude we dubbed an “humanitarian worldview”.^3^ The twentieth Century had seen a growth in humanitarian attitudes toward most deviant groups in Western societies and it seems that it was this cultural trend, rather than endorsement of the disease concept, that largely accounted for positive historical change in attitudes to alcoholics. Whether or not one believed that alcoholics deserved treatment and that public funds should be devoted to it was much better predicted by one’s political affiliation and what newspaper one read than by whether or not one publicly endorsed the disease concept of alcoholism. It would be fascinating to try to replicate these findings today and with different addictions.

Other surveys in the older literature also produced findings inconsistent with the equation of disease beliefs and sympathetic attitudes to alcohol addicts (see Heather and Robertson [7, pp. 100–101]) and cast doubt on Leshner’s [9] assumption that belief in the disease concept and belief that addicts are morally weak are incompatible positions. Although several studies showed that the disease concept had been successfully implanted in the public mind, a corresponding decline in moral attitudes had not occurred. For example, as early as 1964 Mulford and Miller [22] reported that 75 % of their sample defined alcoholics as “morally weak” or “weak-willed” while 41 % defined them as both morally weak and ill. In other studies, endorsement of moral weakness statements was more common in respondents who endorsed a disease conception than in those who rejected it [7, 21]. All this evidence suggests that acceptance of the disease concept of addiction by the general public is largely lip-service.

### Stigma and the BDMA

More recently, Bell and colleagues [23] found very mixed support for the BDMA among clinicians involved in the treatment of addictions in Australia, echoing earlier findings of ambiguous and ambivalent attitudes to the disease concept of alcoholism among clinicians in the UK [see 7, p. 101). Although more research of this kind would be valuable, the conclusion must be that the claim that the BDMA removes stigma among professional workers, and is therefore of benefit in the treatment of addiction, is not currently supported by evidence.

Rachel Hammer and colleagues [24, 25] conducted in-depth, semi-structured interviews with 63 patients in treatment for addiction in alcohol and/or nicotine treatment centres in the US Midwest, as well as 20 addiction scientists of various kinds. Interviewees were asked about their understanding of addiction, including whether they considered it to be a disease. The authors’ conclusion from these data was that, despite popular arguments that framing addiction as a disease will improve treatments outcomes and decrease moral stigma, such a framing is not only unnecessary but may actually be harmful. They also pithily observe: “Rather than a malady of the weak-willed, addiction reframed as a pathology of the weak-brained (or weak-gened) bears just as much potential for wielding stigma and creating marginalized populations” [25, p.28].

### Stigma and Biogenetic Explanations of Behavioural Disorders in General

The issue of the societal effects of the BDMA and of disease concept of addiction more generally cannot be separated from the wider effects of biogenetic explanations of mental illness and behavioural disorders on the public’s attitudes and responses. After all, addiction attracts a psychiatric diagnosis and is specified in the *Diagnostic and Statistical Manual of Mental Disorders* (DSM), albeit in the fifth and latest manifestation [26] as ‘substance use disorder’.

Kvaale and colleagues [27] carried out the first meta-analytic review of studies looking at the effects on stigma of biogenetic explanations of mental disorders, including substance use disorders. Samples included in the review consisted of lay people, professionals, and individuals themselves affected by psychological problems. The main finding was that biogenetic explanations did appear to reduce blame but also induced pessimism over the future prospects of those suffering from these disorders. It was also found that biogenetic explanations increased endorsement of the stereotype that people with psychological problems are dangerous, an understandable reaction to the idea that addiction, for example, is the result of permanent changes to brain mechanisms over which the sufferer has no control.

The idea that, rather than encouraging sympathetic attitudes, brain disease explanations may have the opposite effect is supported by findings from a study by Mehta and Farina [28]. In a contrived experiment, they asked volunteers to administer either mild or strong electric shocks to two groups of ‘patients’ if they failed a certain test. Patients believed to have a brain disorder were shocked at a higher level and faster rate than those whose disorder was believed to be psychosocial in origin. As the authors say, their results “provide little support for the claim that regarding the mentally disordered as sick or diseased will promote greater acceptance or more favourable treatment” (p. 405). A similar experiment specifically on addiction would be useful.

We know from other research that those among us who are seen as suffering from behavioural abnormalities of biological origin are viewed by the average person as dangerous and unpredictable, resulting in efforts to avoid interacting with them, with the inevitable consequence that the sufferer’s sense of isolation and alienation is exacerbated. As James Davies [29] has put it: “Paradoxically, … the worldwide psychiatric campaigns whose goals are to reduce stigma associated with mental illness by asserting that it’s just like any other biological disease may well have helped bring about the very opposite of what (was) intended” (p. 223).

Nor can it be claimed with any justification that biogenetic explanations increase empathy among clinicians treating behavioural disorders. Lebowitz and Ahn [30] reported a series of studies in which clinicians in the USA read descriptions of potential patients whose symptoms were explained in either biological or psychosocial terms. Rather than increasing clinicians’ empathy (on the ground that patients were less blameworthy for their actions), biological explanations of behaviour evoked significantly less empathy. These results, say the authors, “are consistent with other research and theory that has suggested that biological accounts of psychopathology can exacerbate perceptions of patients as abnormal, distinct from the rest of the population, meriting social exclusion, and even less than fully human” (p. 17786).

Interestingly, a group of psychiatrists has recently written about what they perceive to be a crisis in their science and profession [31]. Although not disparaging the brain sciences and psychopharmacology, they argue that psychiatry needs to move beyond the dominance of what they call the current, technological paradigm, a move that would be more in keeping with evidence about how good outcomes are achieved and might also foster more meaningful collaboration with the growing service user movement. They go on:

“… the promise of therapeutic gains from the brain sciences always seems to be for the future, leading some to interrogate their contribution to advances in our field… Indeed, neuroscientists themselves have become more cautious about the value of reductionist approaches to understanding the nature of human thought, emotion and behaviour… Furthermore, there is ample evidence that anti-stigma campaigns based on biogenetic models of serious mental illness have been counterproductive” (p. 430).

From the evidence briefly reviewed above, there must be a presumption that this counterproductivity of biogenetic models embraces the addictions field. Indeed, the onus now lies with proponents of the BDMA to demonstrate that this is not the case.

## So, if Addiction Is Neither a Brain Disease Nor a Moral Failing, What Is it?

The main purpose of this article has been to show that the BDMA does not necessarily lead to more benign attitudes to addiction among the general public and professional workers. However, the question in the heading above demands an answer before we proceed further, even if that answer is self-evident. As already noted, the model of addiction described by Marc Lewis in his article in this Special Issue is clearly a scientific explanation of addiction that eschews disease and yet does not suggest blaming or punishing addicts.

There are, unsurprisingly, other possibilities. One is to see addiction primarily as a disorder of choice [1, 32, 33]. There will not be space here to discuss these theoretical issues with any thoroughness but a few observations may be made. Such a view of addiction might not be incompatible with Lewis’ developmental-learning model but would place more emphasis than Lewis gives on reinterpreting the role of choice in addiction (see Lewis [3, p. 8]); while Lewis’ model is concerned primarily with how addiction develops, a disorder-of-choice model is concerned to describe and explain the essential nature of addiction as a failure of self-regulation. Addiction is seen as a disorder of choice in the sense that it represents a kind of failure to make consistent choices over time [34]. Thus, although addicts respond to incentives and are free to choose to use or not to use at any one time, autonomy is impaired when their pattern of choices is considered over time (i.e., their ‘extended agency’ [34]). A person makes a strong resolution at time t^1^ to desist from a specified behaviour at time t^2^ but, when t^2^ occurs, fails to carry out that resolution. When that happens repeatedly and distressingly, we can describe this pattern of behavior as addiction [35]. Though addictive behaviour is voluntary at the time it is carried out, addiction involves an interaction of voluntary and involuntary processes conceptualised in a dual-systems account of human behaviour [1]. Without going further (but see [1, 34]) we may note that this understanding of addiction makes central use of the ancient philosophical concept of *akrasia* or ‘weakness of will’ [35], as well as concepts such as temptation, self-control and willpower. So too, addictive behaviour is assumed to be voluntary, not compulsive, at the time it is carried out [36].

It should go without saying that the employment of such terms in a scientific theory of addiction has no logical consequences for whether or not addicts should be blamed and punished; it is possible to say that addicts are ‘responsible’ for their behaviour, in the sense that addictive behaviour is voluntary at the time it is enacted, without at the same time blaming them for it [37]. Nevertheless, such language is anathema to devotees of the BDMA and they would undoubtedly claim that a reversion to punitive attitudes among the public would follow if such a theory became widely known.

It must indeed be conceded that a theory of addiction couched in terms of weakness of will, self-control and willpower would present special difficulties for communication. Misunderstandings would be fuelled by oversimplified, distorted and sensationalist portrayals in the media, including those prompted or taken advantage of by scientists and clinicians with vested interests in the BDMA. There is also the danger that models allowing some role for choice in addiction would play into the hands of those who profit from the sale of products with addiction potential, especially those in the smoking, alcohol and gambling industries who appeal to the consumer’s “freedom to choose” and their “personal responsibility” for controlling their consumption. Such discourses would also be endorsed by governments of a neoliberal complexion. Thus it would be naïve not to recognize these societal and political risks of proposing that addiction is a disorder of choice.

## How Could Addiction as a Disorder of Choice Be Presented to the Public?

It may be true that weakness of will or, in other language, ‘above average difficulty in behavioural self-regulation’ is a characterological or personality trait that is evident across a wide range of situations and persists over long periods of time. If so, however, that would be less relevant to a model of addiction as a disorder of choice than another aspect of weakness of will – that *we are all weak-willed*. That is to say, addicts struggle with extreme variants of a difficulty in controlling behavior that affects all members of the human race past infancy on a daily basis and has been recognised at least since the story of Adam and Eve as a fundamental aspect of the human predicament. If empirical evidence for this assertion is needed, Hoffman and colleagues [38] studied a large sample of adult citizens of Germany and found that people reported fighting against a desire for approximately one-quarter of their waking hours, giving in on roughly half of occasions. So too, when people in the USA were asked about reasons for failing to meet their goals for healthy living, ‘lack of willpower’ was named as the most important factor [39].

All this suggests that, if addiction were presented to the public as an extreme version of a problem with which we are all familiar— and buttressed perhaps by slogans along the lines of, “There but for the grace of God go I” - understanding and compassion might be increased and stigma avoided or, at least, reduced. There has been a recent flurry of self- help books on willpower aimed at the popular market [40–43] and, assuming that these books achieve some commercial success, this suggests that the public might be receptive to such messages. This kind of education would have the opposite effect to telling people that addicts have a mysterious brain disease. Communications about addiction as a disorder of choice would emphasise the continuity of the experience of ‘addicts’ with that of people not so labelled and the intelligibility of addictive behaviour and experience to ordinary people. This would contrast sharply with communications about addiction as a disease based on some ‘pathological’ process applying only to addicts that the layperson finds unfamiliar and unintelligible but is expected to take on trust.

A remaining objection from supporters of the BDMA can be anticipated. This is that, even if the weakness of will component of a disorder-of-choice model of addiction were understood by the public, there would remain the problem that addictive behaviour was described in this model as involving voluntary behaviour – or what Volkow and Li [44] would prefer to call “the pernicious yet enduring popular belief that their (addicted individuals’) affliction stems from voluntary behaviour” (p.1430, parentheses added). As such, they would claim, there would be nothing to prevent addicts from facing continued stigmatisation. It is, of course, meaningless to say that addiction ‘stems’ from voluntary behaviour but what is true is that the disorder-of-choice model posits that addiction involves an interaction of voluntary and involuntary processes. This is not the place to enter into the complexities of the debate as to whether, and if so in what way, addictive behaviour can justifiably be described as compulsive (but see [45]). However, arguably one reason the disease view may not be accepted by some members of the public (and some academics) is that drug-seeking and drug-taking, by an addict or by anyone else, is self-evidently voluntary behaviour and, further, that to deny this palpable fact is an outstanding example of the emperor’s new clothes [46]. If so, what is needed is a model that continues to see addiction as behaviour that people find extremely difficult to change while at the same time accepting the obvious fact of voluntary drug-seeking and –taking. A model of this kind would be to see addiction as a disorder of choice.

## Addiction and Access to Treatment

Marc Lewis [3] makes a good case for the scientific inadequacy of the BDMA and others too have demonstrated its limitations [e.g. 47–49]. However, one response to these criticisms is to concede the deficiencies of the BDMA, or some of them, but to maintain that the disease concept is still necessary in public-facing communications to ensure that addicts get access to treatment, insurance coverage, time off from work and so forth, as well as persuading governments to fund research and rehabilitation facilities, etc. [50, p.461]. The brain disease version of the disease concept would simply be the most current and authoritative of possible disease concepts available for this purpose. While the underlying ‘pathology’ of the disease of addiction is yet to be demonstrated, so this argument would run, there is no consensus on the definition of disease and so, because ‘treatment’ is obviously a medical term, calling addiction a disease is necessary to get addicts a better deal from society than they would otherwise receive. Many years ago Robin Room [51, p. 1056] called this argument “humane cynicism”.

Is it true that advancing an understanding of addiction as a disorder of choice or a developmental-learning disorder, assuming they were widely accepted, would decrease access to treatment and all the other alleged societal benefits of the BDMA? Again, it is easy to see how misunderstandings might arise. A solution to this particular problem, however, would be to emphasise that addictive behaviours are typically health-damaging behaviours and therefore need to be ‘treated’ in medical settings in order to prevent or limit the progress of diseases that are undoubtedly of legitimate medical concern.^4^ This already happens to some extent in the deployment of brief interventions in medical settings against smoking, excessive alcohol consumption, over-eating and other health-damaging behaviours [52]. All that is needed is to regard addiction as continuous throughout the population of regular users of psychoactive substances and to extend the principle of intervention to the treatment of addictive behaviours in general. Indeed, one might go further and argue that the concept of addiction would become theoretically redundant if addictive behaviours were subsumed under the larger set of hard-to-reduce/eliminate health-damaging behaviours [14, 53]. Be that as it may, there should be little difficulty in obtaining access to treatment, insurance coverage and research funding for secondary and tertiary prevention of diseases caused by addictive behaviours.

## The Future of Addiction

Marc Lewis ends his essay in this Special Issue [3] with some inspiring passages concerning the need for “a third stage” in our collective understanding of addiction. The first stage, beginning in pre-industrial times and still influential in some quarters today, was the moral understanding in which what we would now call addicts were held to be morally responsible for their behaviour and the appropriate response was “to punish the addict through scorn, isolation, disenfranchisement, or incarceration” (p. 15). The second stage, beginning in the early nineteenth Century and culminating in today’s BDMA, is the disease understanding in which “the appropriate solution to addiction is to be found in the realm of medicine (and in which) … addicts should be urged (convinced or compelled) to follow the advice handed down by medical practitioners” (p. 16). Lewis calls for a third stage based on the developmental-learning model outlined in his essay in which recovery is seen in terms of individual development and growth beyond addiction.

Lewis and I differ on the details of what this third stage in the understanding of addiction should look like but I agree with him wholeheartedly on the need for it and on the need, in particular, to shake off the stultifying dominance of the BDMA in addiction science and practice. What worries me most about the BDMA is that, despite the pious concessions of its proponents about the need to take into account social ‘factors’, by far its major practical consequence is the attempt, in the unholy collaboration between medicine and the pharmaceutical industry, to invent yet more psychoactive substances and other medical procedures to correct the purported deficiencies in brain functioning held to be responsible for the disease of addiction [54]. Rather than resulting in any miracle cure, this is only likely to replicate the catastrophic failure of drug therapy for mental illness that has occurred over the last 70 years [55] and perpetuate the over-medicalisation of problems in living that others have warned us about [56]. No sensible person would dispute the potential contribution of neuroscience to our understanding of addiction but, to my mind, the BDMA is not only inhumane, it is also deeply unintelligent in its ‘eliminative reductionism’ [57] and its resulting inability to begin to grasp what addiction is about. We need a new ‘governing image’ [58] in public discourse about addiction and we need thinkers like Marc Lewis to help show us the way.

## Footnotes

1 Another neuroscientist who rejects the brain disease model of addiction is Carl Hart [2].

2 Visit http://www.memoirsofanaddictedbrain.com/media-biology-of-desire/ for a list of articles, reviews, interviews etc. regarding Marc Lewis’ book, *The Biology of Desire* (Marc Lewis, 2017, Addiction and the brain: development, not disease, Neuroethics), .

3 An earlier study by John Crawford looked at this issue from a different perspective. In a comparison of attitudes toward alcoholics and compulsive gamblers, he found that, although there was a large difference in the extent to which members of the public saw these conditions as a disease or illness, they did not differ in the extent to which they considered alcoholics or compulsive gamblers to be entitled to treatment. These findings illustrate the idea that the disease label need not convey an advantage in the attempt to engender positive attitudes to the treatment of addicts. See Crawford and Heather [21].

4 This is not to say that the damaging consequences of addictive behaviours are restricted to health; they are obviously far wider than that. The point here, however, is that, within a medical context, emphasising the health-damaging consequences of addictive behaviours legitimises their place within health services without the need to define the behaviours themselves as diseases.
